# Re-examination of the mechanical anisotropy of porcine thoracic aorta by uniaxial tensile tests

**DOI:** 10.1186/s12938-016-0279-6

**Published:** 2016-12-28

**Authors:** Qiang Chen, Yan Wang, Zhi-Yong Li

**Affiliations:** 1 Biomechanics Laboratory, School of Biological Science and Medical Engineering, Southeast University, Nanjing, 210096 People’s Republic of China; 2 School of Chemistry, Physics and Mechanical Engineering, Queensland University of Technology, Brisbane, Australia

**Keywords:** Porcine thoracic aorta, Anisotropy, Young’s modulus, Ultimate stress, Relaxation

## Abstract

**Objective:**

Considering past studies on the orthotropic anisotropy of arteries in the circumferential and axial directions, this work aims to experimentally study the anisotropic behaviour of arteries by tensioning multi-directional strips of porcine thoracic aorta.

**Methods:**

Histology is first analyzed by staining arterial sections of three orthotropic (axial, circumferential, and radial) planes. 168 stripped samples from 21 aortas are categorized into three loading-rate groups to investigate the influence of loading rates on the Young’s modulus and ultimate stress. Basing on the Young’s modulus and ultimate stress, the degree of anisotropy is calculated. Moreover, 24 stripped samples from 3 aortas are tested to study the relaxation anisotropy of arteries by fitting the experimental data with a five-parameter Maxwell reduced relaxation function.

**Results:**

Histological analysis shows the parallel orientation of crimpled collagen and elastin fibres. The Young’s modulus and ultimate stress reach the greatest in the circumferential direction, and the smallest in the axial direction, respectively, and the values in the other directions are in-between; moreover, the two parameters monotonously increase as the samples orientate from the axial to circumferential directions. The Young’s modulus is more sensitive to the loading rate than the ultimate stress. The degree of anisotropy calculated by the Young's modulus is similar to that by the ultimate stress, and it is independent of loading rates. Stress-relaxation also exhibits anisotropy, whose variation is consistent with those of the two parameters.

**Conclusions:**

Due to the stress-growth rule, fibre preferably orientates in the circumferential direction, and the preferable orientation results in great mechanical parameters, anisotropy, and small relaxation behaviour of arteries. The work extends the studies on the arterial anisotropy instead of only the circumferential and axial directions, and could be useful to comprehensively understand the anisotropy of arteries.

## Background

Artery is composed of three layers, i.e., adventitia, media, intima, and is a composite mainly containing three components, i.e., collagen fibre, elastin fibre, and arterial smooth muscle, which determine the mechanical behaviour of arteries. There is a huge amount of literature on the biomechanics of arteries, and the mechanical anisotropy of arteries is one of the classical and fundamental issues. This is because knowing the mechanical anisotropy is very important in improving arterial material models, revealing the remodelling process of arteries, and differentiating normal and abnormal arteries.

Early work experimentally measured the mechanical anisotropy of arteries by different methods [1], such as the uniaxial [2] and biaxial tensile tests [3], physiologically-simulated test (cylindrical arterial segments subjected to an internal pressure and axial pre-stretch without torsion and shear [4]), digital image correlation (DIC) technology [5]. Recently, there are other developed methods to test the arterial anisotropy, for example, non-invasive supersonic shear wave imaging technology [6]. The classical uniaxial and biaxial tensile experiments are easily to perform, and the results are intuitively understandable. However, the uniaxial tensile method only obtains the mechanical behaviour of arteries in one direction at a time, and the biaxial obtains those in two directions interested, moreover, the residual stress is released and thus neglected due to the opening of arterial samples. The physiologically-simulated inflating method approaches the in vivo loading state of arteries, and the residual stress is retained, but it is not easily to capture the arterial anisotropy in other directions except the circumferential and axial directions. Regarding the non-invasive ultrasound-based technology, the acquired data must be transferred into the readable mechanical data, and a reliable algorithm has to be established and verified, in this regard, it is not so convenient. In any case, the uniaxial tensile test as a simple and effective method, although it has some disadvantages, it is still widely employed to study the relevant mechanical issues including the anisotropy of arteries [7–9].

The microstructure-mechanical relationship has been experimentally examined, and theoretical frameworks of arterial anisotropy including fibre orientations have been well developed. As stated in the first paragraph, the arterial microstructure is a three-layered composite with three main components, and the study on the nonlinearity and anisotropy is mainly at the tissue level including the anisotropic failure [10–12]. Regarding the individual contribution of each component, the smooth muscle cell is conventionally assumed to be circumferentially orientated in the 2D plane of arterial walls [13], and thus it contributes most the mechanical response in that direction, however, the recent work [14] reported that the smooth muscle cell is right-handed helically dispersed in 3D by a quantification method, and this result is consistent with the anisotropic model presented by Holzapfel et al. [15], which treated the arterial wall as a two-layered woven structure and further improved the structural model by incorporating more real factors, such as the hyper-elasticity [16] and non-symmetric fibre orientation [17]. Moreover, according to the Hill’s muscle contraction mechanism, sliding movement between the actin and myosin filaments and unfolding the protein domain result in a visco-elastic mechanical behaviour at the molecular level, and adaptively plasticity at the tissue level; plus, due to the visco-elasticity and plasticity, the smooth muscle is believed to strongly influence the relaxation behaviour of arteries [18]. Collagen and elastin play an important role in the nonlinearity and anisotropy of arteries. Gundiah et al. [19] studied their individual and combined contributions on the arterial biomechanics by an enzymatic method, but they did not see any anisotropy in the mechanical response of elastin. This is totally different from the conclusion in [20], which divided the mechanical behaviour of the elastin into isotropic and anisotropic parts, and the result by Agrawal et al. [21], which quantified the regional (from the proximal end to distal end) variations of the anisotropy and nonlinearity of elastin isolated from bovine aorta. Although the above-mentioned work and other unmentioned literature have made considerable progress in the study of the arterial biomechanics, for the anisotropy, most of them still considered the simple orthotropic anisotropy in the circumferential and axial directions.

In this work, by employing the simple but effective uniaxial tensile method and treating multi-directional stripped samples of aorta, the Young's modulus, ultimate stress, the degree of anisotropy, and the relaxation anisotropy are examined and discussed by combining the histological analysis on the collagen and elastin fibres.

## Methods

Twenty-four arteries of adult boars were collected from the Hushu abattoir in Nanjing, China. The arteries were warped by phosphate buffered saline-soaked gauze immediately when they were extracted from animal bodies. After transported to the laboratory, they were preserved in a freezer under the condition of −20 °C for subsequent mechanical experiments within 22 days, because the cryopreservation did not change the important elastic properties of arteries [22].

### Histology

**Fig. 1:**
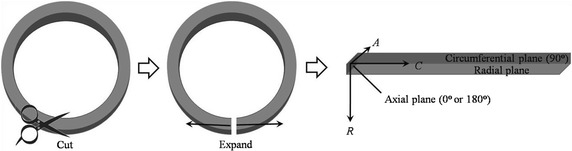
Diagram of expanding a cylindrical arterial segment and definition of three orthotropic planes

The histology aimed to observe distributions of the collagen and elastin fibres in three orthogonal planes (i.e., axial, radial and circumferential planes in Fig. 1), and to correlate them to the mechanical anisotropy of the artery. A cylindrical arterial segment from a fatty-tissue-cleaned aorta, which was unfrozen at room temperature, was first cut open along its axis, and expanded into a flat, then, the expanded artery was divided into three, which were then embed into three paraffin blocks. Furthermore, a microtome (RM2016, Leica, Germany) was employed to obtain three paraffin sections with thickness 8 μm, then, the paraffin sections were dewaxed and examined by an upright microscope (Eclipse CI, Nikon, Japan) equipped with an imaging system (DS-U3, Nikon, Japan). Here, to visualize the collagen and elastin in the three orthogonal planes, the three paraffin sections were stained by the classical Weigert’s resorcin fuchsin method.

### Anisotropy

In this part, two sets of experiments were performed: one was testing the anisotropic stress–strain response to study the influence of the loading rate on the Young’s modulus, ultimate stress and degree of anisotropy; the other was testing the relaxation anisotropy. All samples were uniaxially tensioned by an Instron single column tabletop testing system (5943, Instron, USA).

#### Stress–strain response

**Fig. 2:**
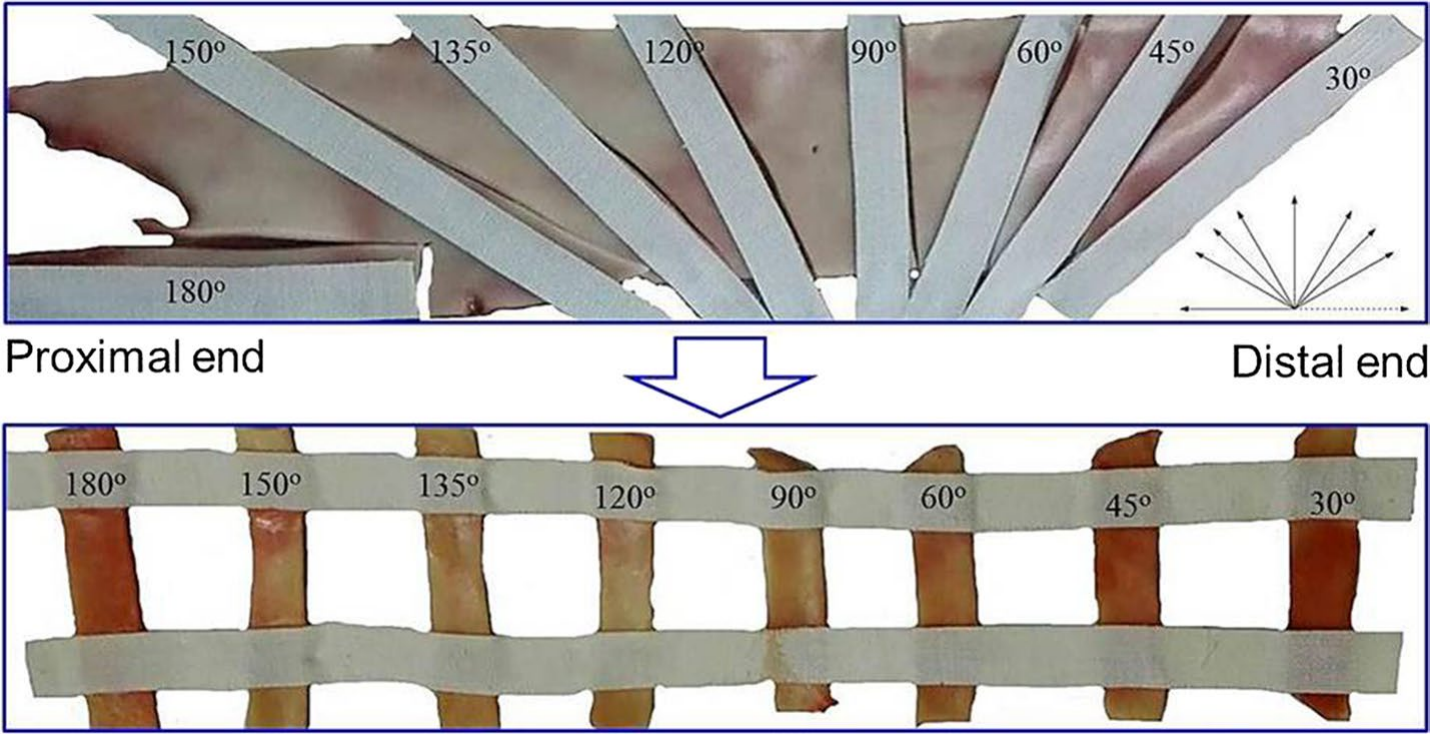
Preparation of the stripped aortic samples with different orientations

Twenty-one arteries were tested in this experiment. For each aorta, like the histological analysis, it was unfrozen at room temperature, and fatty-tissue-cleaned, and then cut open, and further expanded into a flat. Setting the axial direction (from the proximal end to the distal end) of the aorta as 0°, and employing a protractor and adhesive tape to measure and fix the samples, it was anti-clockwisely cut into eight stripped samples with 30°, 45°, 60°, 90°, 120°, 135°, 150°, and 180° (Fig. 2), thus, the total number of stripped samples was 168, and their width and thickness were 10.01 ± 0.18 mm and 1.99 ± 0.26 mm, respectively. All the samples were soaked in the phosphate buffered saline for the mechanical tests. In the tests, the engineering stress *σ* and strain *ε* were calculated by *σ* = *F*/*A* and *ε* = *Δl*/*l*, respectively, where *F* is the tension force, *A* is the initial cross-sectional area obtained by averaging three measurements at two ends and middle position of each stripped sample, *Δl* is the elongation of the samples, and *l* is the initial sample length (i.e., the length between two clamps).

To test the influence of different loading rates on the Young's modulus, ultimate stress and degree of anisotropy, the twenty-one porcine thoracic aortas were categorized into three groups (seven aortas in each group), and for the three groups, they were tensioned in loading rates of 1, 5 and 10 mm/min, respectively. Moreover, the Young's modulus and ultimate stress were obtained to calculate the degree of anisotropy.

#### Relaxation behaviour

**Fig. 3:**
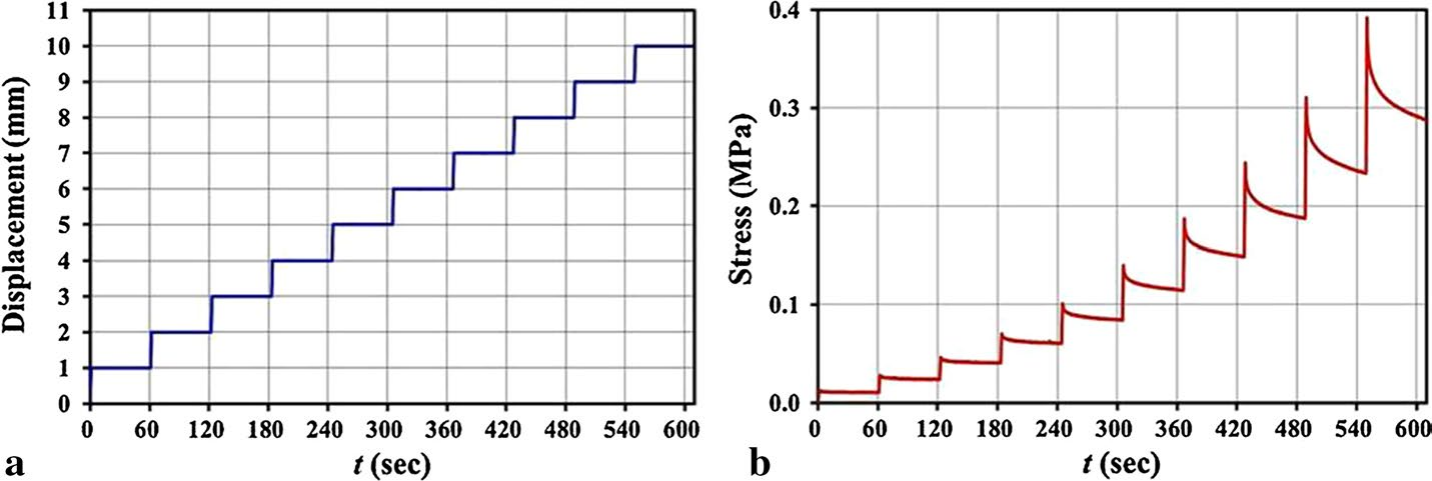
Loading condition (**a**) and typical stress-time response (**b**) of a stripped sample

Three aortas were used to investigate the relaxation anisotropy. Similar as the “Stress–strain response” section, eight stripped samples were made from each aorta, and the total number of stripped samples was 24. Their width was 8.56 ± 0.64 mm, and thickness 1.31 ± 0.26 mm. For all the samples, within the first 95 s, they were cyclically loaded-unloaded five times to eliminate uncertainties produced by the samples themselves or the loading system at the very beginning (this part is not shown in the loading curves), and more, to ensure no slippage, the clamps with rough surfaces were used. After the cyclically loading process, samples were tensioned 1 mm displacement increment within 1 s, then maintained at the constant displacement for 60 s; next, the second 1 mm displacement increment was stretched and another period of 60 s was held; the stretch-maintain process was repeated ten times, as shown in Fig. 3a [23], and the typical stress-time response calculated from the corresponding force–time response recorded by the testing machine was reported in Fig. 3b.

For the relaxation behaviour, the linear visco-elastic constitutive model based on Boltzmann’s superposition principle was extensively cited to describe the mechanical behaviour of biological soft tissues, and it is written as [24]:

1 $$ \sigma (t) = \int_{ - \infty }^{t} {G(t - \tau )} E^{(e)} \frac{\partial \varepsilon (t)}{\partial t}{\text{d}}\tau $$

where *σ*(*t*) is the tensile stress, *G*(*t* − *τ*) is the relaxation function, *E* ^(*e*)^ is the instantaneous Young’s modulus, which is determined by fitting a line to the experimental instantaneous stress–strain curve, *ɛ*(*t*) is the tensile engineering strain. According to Iatridis et al. [23], a five-parameter discrete Maxwell solid model here was employed to define the relaxation function:

2 $$ G(t) = \frac{{G_{0} + (\mu_{1} /\tau_{1} )e^{{ - t/\tau_{1} }} + (\mu_{2} /\tau_{2} )e^{{ - t/\tau_{2} }} }}{{G_{0} + \mu_{1} /\tau_{1} + \mu_{2} /\tau_{2} }} $$

where *G*_0_ is a fitting parameter related to the normalized elastic constant, and *μ*_1_ and *μ*_2_ define the viscosities corresponding to time constants *τ*_1_ and *τ*_2_, respectively.

#### Estimation of parameters

**Fig. 4:**
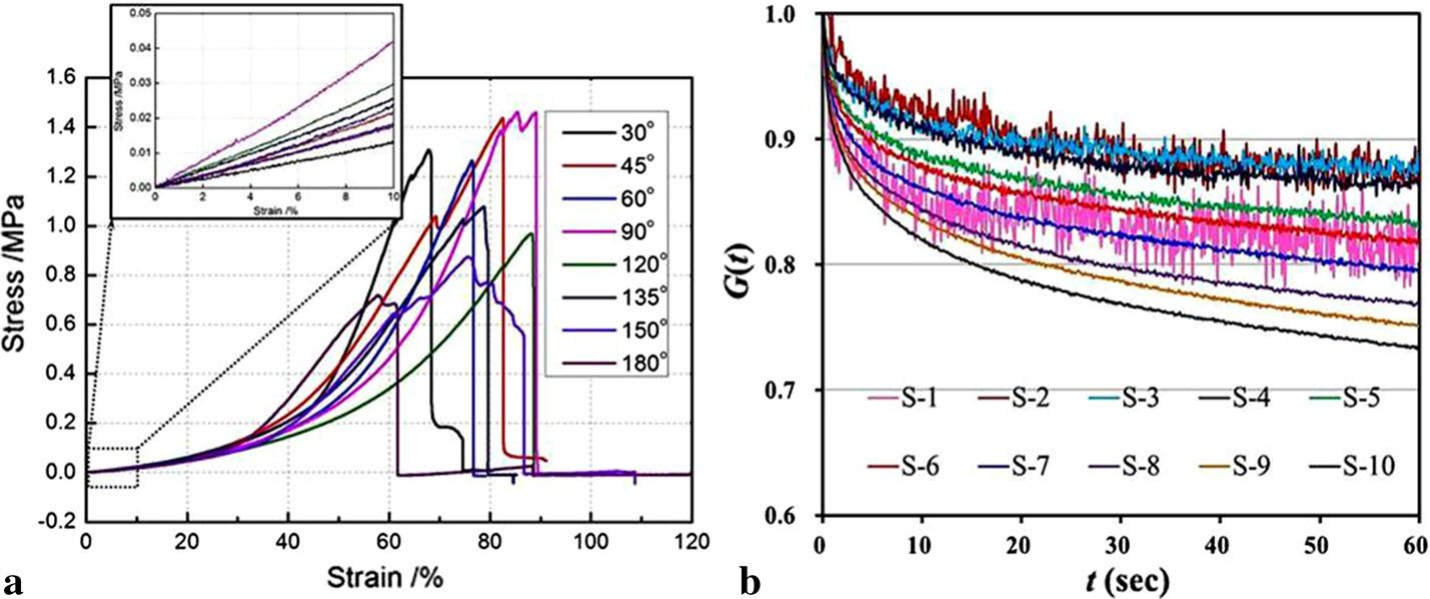
Typical stress–strain curves of a set of samples (**a**) and a reduced relaxation function *G*(*t*) of ten steps (or at ten stress levels) of a 30° sample (**b**) from an aorta

For the Young’s modulus, according to Lillie et al. [25], which used the inflation method to test the mechanical anisotropy of pig aorta, and the Young’s moduli of the circumferential and axial directions were nearly linear before the strain 10%, thus, here we also employed the portion (strain ≤10%) of stress–strain curves to calculate the samples’ Young’s moduli (insets in Fig. 4a). Both the Young’s moduli and ultimate stress of the 168 samples were statistized in the form of box-plot. Moreover, the degree of anisotropy *λ*_*θ*_ calculated by the mean values of the Young’s modulus and ultimate stress were defined as $\lambda_{\theta } = {\bar{M}_{\theta } } /{\bar{M}_{{90^{\text{o}}}}}$, where *θ* is the orientation of stripped samples, $\bar{M}_{\theta }$ is the mechanical properties of the stripped samples, $\bar{M}_{{90^{\text{o}} }}$ is the mechanical properties of the circumferential stripped sample. This parameter is an important index and often used to describe the anisotropy of materials [25].

The reduced relaxation function *G*(*t*) was calculated through normalizing the stress at each step holding stage (0 < *t* < 60 s) by its peak stress (*t* = 0), which is the stress data point when the holding stage initiated, i.e., $G(t) = {\sigma_{i} (t)}  / {\sigma_{i} (0)}$. (*i* = 1,…,10) [23], where *t* is the holding time in each step instead of the time in the whole stretch-maintain process, and *i* denotes the step number. The defined function indicated the relaxation ability at different stress levels *σ*_*i*_(0), see Fig. 4b, in which S-i denotes the *i*th step. Then, for the 24 samples (from the three aortas) at the same orientation and stress level, the experimental data were averaged. Employing the least squares regression, the averaged experimental data were fitted by the Maxwell relaxation model [Eq. (2)] to obtain sets of parameters (*G*_0_, *μ*_1_, *μ*_2_, *τ*_1_, *τ*_2_), and more, the fitted parameters of all stress levels of each orientational sample were averaged. Finally, using the averaged fitted parameters, the reduced relaxation functions *G*(*t*) of different orientational samples were plotted.

## Results

### Histological analysis

**Fig. 5:**
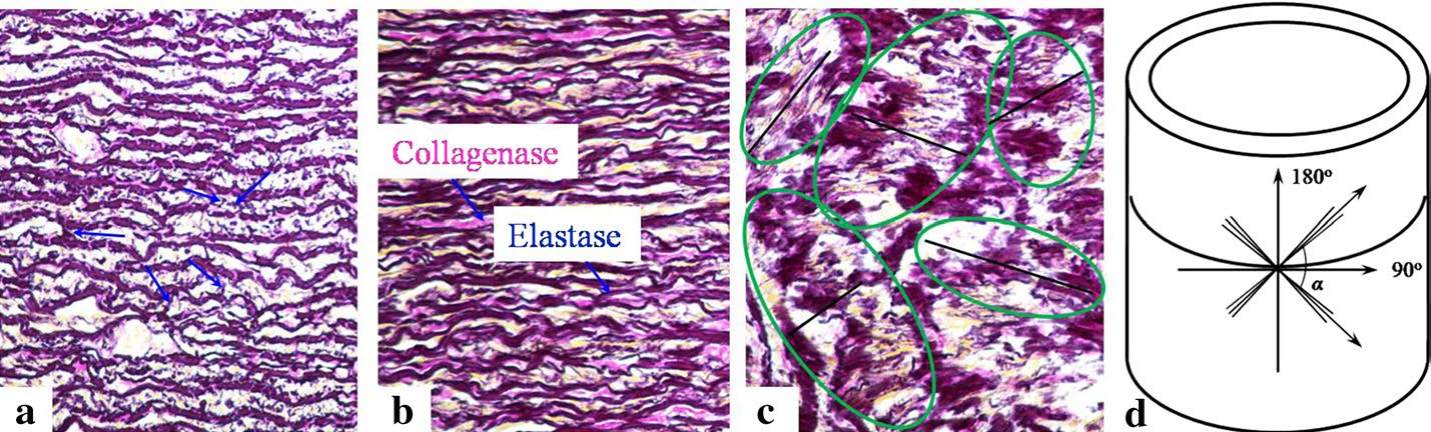
Weigert’s resorcin fuchsin stained **a** axial, **b** circumferential, **c** radial sections from an arterial segment, **d** anisotropic woven model of arterial walls [16]

The microstructure of the crimpled collagen (red) and elastin (dark blue) fibres in the three stained orthogonal planes of an artery are clearly shown in Fig. 5. The collagen and elastin fibres are roughly parallel, and the different fibre sub-layers are connected by inter-layer fibres (blue arrows in Fig. 5a) to form a spatial network. The fibres in the circumferential plane seem much denser arranged than those in the axial one (Fig. 5a, b), and this enables the circumferential samples with a better mechanical behaviour shown in the next section and the degree of anisotropy. Moreover, Fig. 5c shows that the fibres generally form cross-linked network in two directions (the black lines in the regions circled by green ellipses), and this is like the woven model of arteries presented by Gasser et al. [16] (Fig. 5d). The two main orientations in the woven model were also verified by statistizing the orientations of the collagen and elastin fibres of inner adventitia at the zero-stress state [26].

### Anisotropic analysis

#### Mechanical properties

**Fig. 6:**
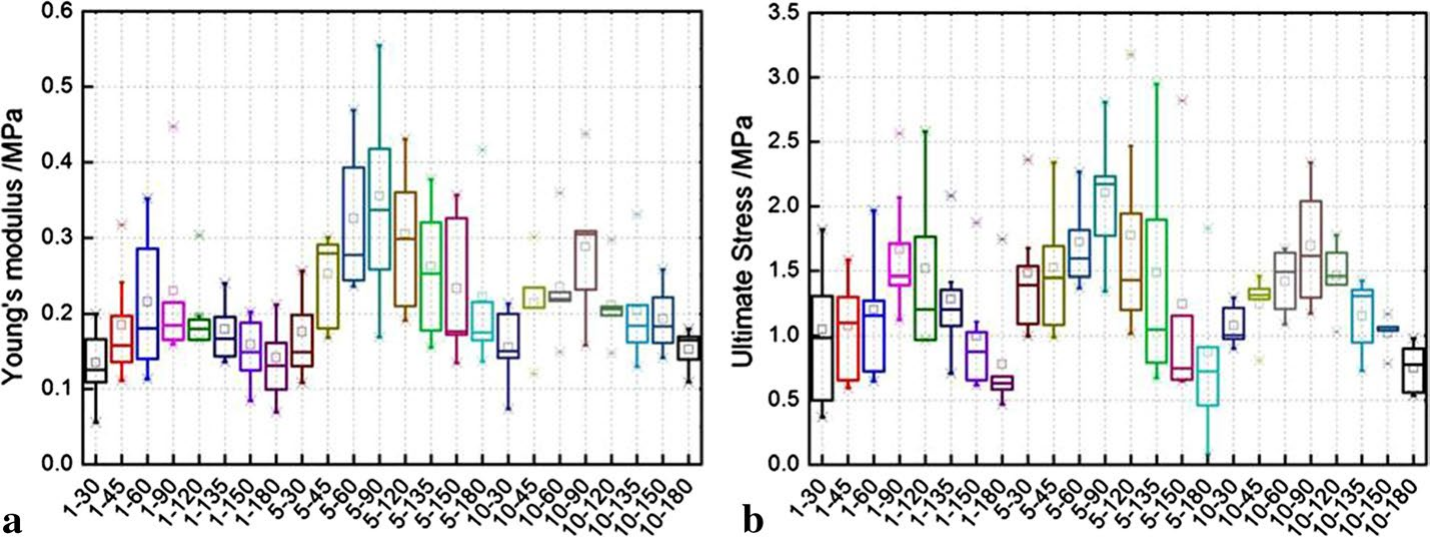
Influences of loading rates on the **a** Young’s modulus and **b** ultimate stress. *Note* the abscissa scale is displayed in the form of *p*–*q* the first number *p* represents loading rate (mm/min), and the second *q* represents samples’ angle (°)

The Young’s modulus and ultimate stress of the samples are plotted in Fig. 6. Compared to the ultimate stress, the loading rate produces a greater effect on the Young’s modulus, in other words, the Young’s modulus is loading-rate-dependent, but generally they are in the range of 0.1–0.4 MPa, and this is comparable to the value (~0.4 MPa at 10% strain level) obtained by the inflation method [25]. Moreover, the Young’s modulus is optimized at the loading rate 5 mm/min with respect to their counterparts at the other loading rates, but its degree of dispersion is higher. For each loading-rate case, both the Young’s modulus and ultimate stress reach the greatest and smallest at 90° and 180°, respectively, and this is related to the denser and looser fibre distributions at 90° and 180° mentioned in “Histological analysis” section, which leads to the arterial anisotropy. This is also consistent with literature, where the circumferential (90°) and axial (180°) samples were only reported [20, 27]. What is more, the two mechanical parameters exhibit a common quasi-sinusoidal variation, namely, they gradually increase from the 30° to 90° samples, and decreases from the 90° to 180° samples.

**Fig. 7:**
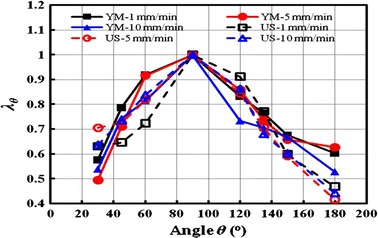
Degree of anisotropy calculated by Young’s modulus (*YM*) and ultimate stress (*US*)

Different from the Fig. 6 presented all the absolute values of samples’ mechanical parameters, the degree of anisotropy, which is more intuitively to show the arterial anisotropy, is calculated and plotted in Fig. 7. It is easily seen that the degree of anisotropy is not distinct, and this indicates that the degree is not much influenced by the loading rate, and the degrees calculated by both Young’s modulus (YM, connected solid markers) and ultimate stress (US, connected empty markers) are feasible.

#### Relaxation

**Fig. 8:**
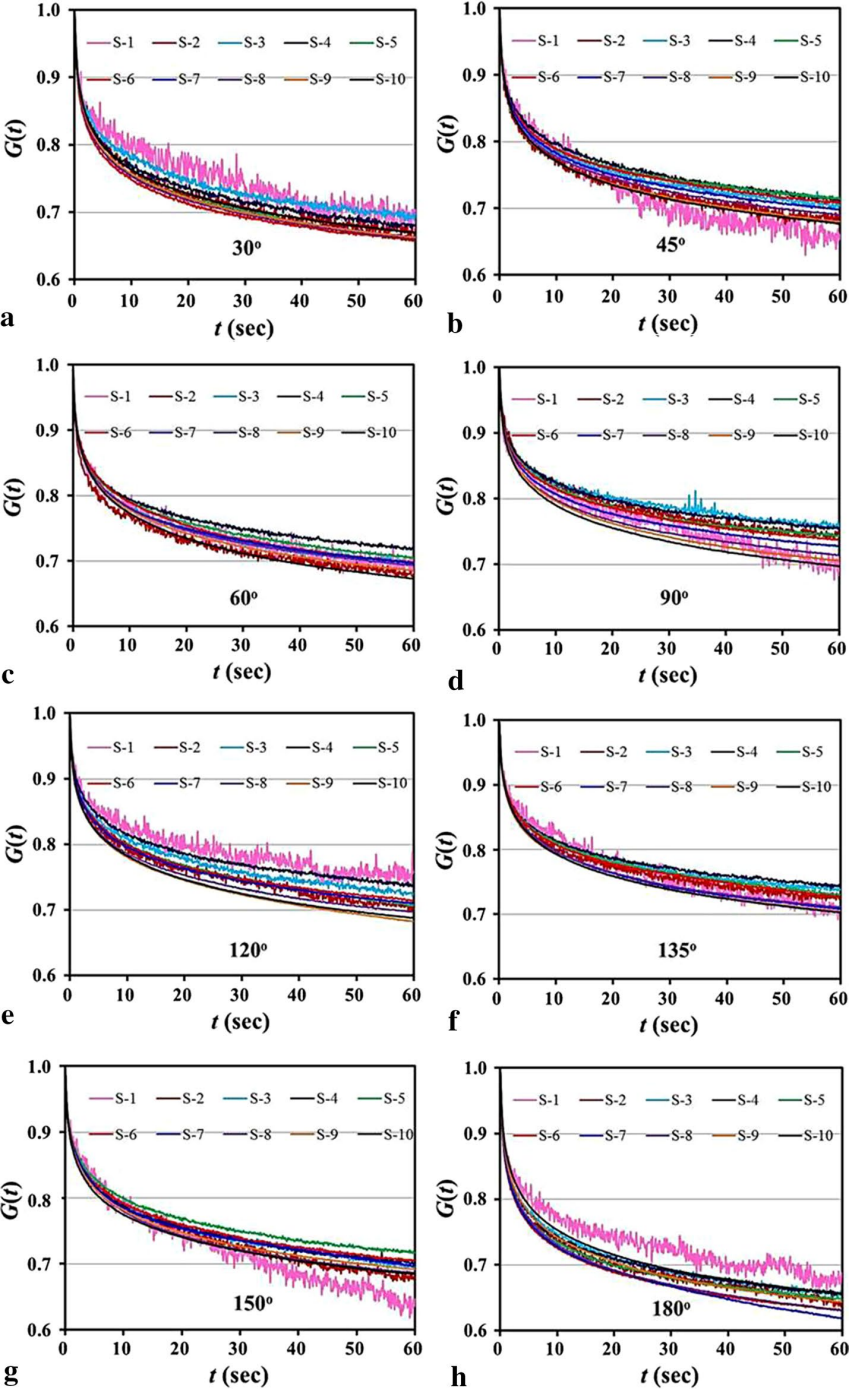
Averaged experimental data of all the samples at the same orientation and stress level. **a** 30º samples; **b** 45º samples; **c** 60º samples; **d** 90º samples; **e** 120º samples; **f** 135º samples; **g** 150º samples; **h** 180º samples

The experimental data of the eight orientational samples at the same orientation and stress level are averaged and plotted in Fig. 8. The normalized reduced relaxation curves generally decay to 0.7, and it seems that there is no big difference between the multi-orientational samples. The higher stress level leads to a greater stress relaxation (clearly seen in Fig. 4b), and a lower stress relaxation occurs at the stress levels from the second step (S-2) to the fourth step (S-4) except the 30°, 120° and 180° samples (Fig. 8a, e, h).

**Fig. 9:**
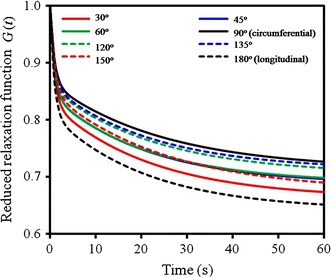
Comparison of the predicted reduced relaxation functions of the samples with different orientations

Correspondingly, the fitted parameters (*G*_0_, *μ*_1_, *μ*_2_, *τ*_1_, *τ*_2_) to the experimental data in Fig. 8 are listed in Table 1. As stated in “Estimation of parameters” section, the mean values ($\bar{G}_{0}$, $\bar{\mu }_{1}$, $\bar{\mu }_{2}$, $\bar{\tau }_{1}$, $\bar{\tau }_{2}$) of the eight orientational samples are obtained by averaging their fitted parameters of all stress levels. Substituting the mean values ($\bar{G}_{0}$, $\bar{\mu }_{1}$, $\bar{\mu }_{2}$, $\bar{\tau }_{1}$, $\bar{\tau }_{2}$) into the relaxation function Eq. (2), the reduced relaxation curves of the samples at different orientations are plotted in Fig. 9. It shows that the relaxation of the 90° (or circumferential) sample is lowest, and that of the 180° (or axial) sample is highest. In this regard, again, it is related to the fibre distributions at 90° and 180° (see “Histological analysis” section). As for the samples at the other orientations, they are in-between.

**Table 1:** Parameters of the five-parameter Maxwell visco-elastic model by fitting the averaged experimental data in Fig. 8

Angle	Parameters	Ten steps											
		S-1	S-2	S-3	S-4	S-5	S-6	S-7	S-8	S-9	S-10	Mean	SD
30°	*G*_0_	0.79	0.84	0.86	0.78	0.74	0.81	0.78	0.77	0.78	0.78	0.792	0.033
	*μ*_1_	0.14	0.17	0.16	0.20	0.21	0.19	0.20	0.20	0.20	0.20	0.185	0.021
	*τ*_1_	0.79	0.82	0.83	0.99	0.99	0.85	0.95	0.96	0.96	0.99	0.913	0.076
	*μ*_2_	7.62	3.83	3.95	4.10	4.69	3.73	3.96	3.96	4.05	4.07	4.395	1.102
	*τ*_2_	33.09	17.94	20.28	22.77	24.56	18.58	20.28	20.19	20.25	20.92	21.885	4.141
45°	*G*_0_	0.83	0.81	0.86	0.86	0.88	0.84	0.84	0.82	0.80	0.79	0.833	0.026
	*μ*_1_	0.12	0.18	0.17	0.16	0.16	0.17	0.17	0.18	0.18	0.19	0.169	0.017
	*τ*_1_	0.76	0.90	0.86	0.89	0.86	0.93	0.92	0.92	0.93	0.98	0.894	0.056
	*μ*_2_	6.31	4.14	3.57	3.95	3.21	3.73	3.75	3.99	4.07	4.11	4.084	0.792
	*τ*_2_	21.69	22.04	20.99	22.45	19.47	21.90	20.74	21.64	21.32	21.06	21.330	0.795
60°	*G*_0_	0.81	0.84	0.82	0.85	0.83	0.86	0.82	0.83	0.80	0.79	0.824	0.020
	*μ*_1_	0.18	0.17	0.18	0.17	0.17	0.15	0.18	0.17	0.19	0.18	0.176	0.009
	*τ*_1_	0.95	0.82	0.95	0.96	0.94	0.86	0.98	0.95	0.99	0.98	0.938	0.053
	*μ*_2_	4.13	3.78	3.64	3.42	4.04	4.24	3.73	3.80	4.11	4.41	3.930	0.289
	*τ*_2_	22.58	20.28	20.77	21.50	22.68	20.87	21.22	20.08	21.53	21.12	21.264	0.816
90°	*G*_0_	0.70	0.86	0.92	0.95	0.95	0.90	0.88	0.86	0.83	0.82	0.868	0.069
	*μ*_1_	0.15	0.17	0.14	0.13	0.13	0.14	0.16	0.16	0.17	0.17	0.152	0.013
	*τ*_1_	0.93	1.12	0.86	0.86	0.82	0.88	0.93	0.94	0.97	0.96	0.926	0.080
	*μ*_2_	12.88	3.61	3.52	3.11	3.21	3.70	3.62	3.79	4.20	4.28	4.592	2.784
	*τ*_2_	52.63	22.86	24.78	19.88	18.88	22.03	21.33	21.27	22.19	22.10	24.796	9.400
120°	*G*_0_	0.88	0.78	0.85	0.89	0.80	0.88	0.85	0.82	0.79	0.81	0.836	0.039
	*μ*_1_	0.15	0.18	0.16	0.15	0.18	0.15	0.16	0.17	0.18	0.17	0.166	0.012
	*τ*_1_	0.99	1.04	1.00	0.96	1.05	0.89	0.94	0.96	0.98	0.95	0.977	0.044
	*μ*_2_	3.88	4.87	3.89	3.53	4.76	3.70	3.84	4.14	4.64	4.32	4.156	0.444
	*τ*_2_	24.50	27.56	22.68	21.50	26.36	19.61	20.92	22.07	23.31	21.68	23.019	2.349
135°	*G*_0_	0.85	0.83	0.92	0.90	0.86	0.84	0.87	0.85	0.84	0.83	0.858	0.029
	*μ*_1_	0.12	0.18	0.14	0.16	0.16	0.17	0.16	0.16	0.17	0.17	0.159	0.016
	*τ*_1_	0.80	1.05	0.86	0.94	1.00	1.05	0.91	0.95	0.95	1.00	0.949	0.076
	*μ*_2_	6.42	3.58	3.18	3.06	3.80	3.83	3.97	3.91	4.04	4.03	3.982	0.877
	*τ*_2_	27.58	22.89	19.47	20.38	23.35	23.96	20.65	21.23	21.80	21.42	22.272	2.207
150°	*G*_0_	0.56	0.76	0.81	0.84	0.88	0.84	0.78	0.82	0.82	0.80	0.791	0.081
	*μ*_1_	0.21	0.16	0.17	0.17	0.15	0.17	0.20	0.17	0.18	0.19	0.177	0.016
	*τ*_1_	1.38	0.93	0.97	0.91	0.89	0.93	1.12	0.95	0.95	1.00	1.003	0.139
	*μ*_2_	12.36	6.49	4.40	4.08	3.79	4.06	4.02	4.04	4.03	4.01	5.127	2.521
	*τ*_2_	45.13	29.01	23.35	22.34	21.05	21.96	23.59	19.98	21.13	21.27	24.882	7.154
180°	*G*_0_	0.80	0.77	0.81	0.79	0.81	0.76	0.67	0.74	0.75	0.77	0.766	0.039
	*μ*_1_	0.16	0.20	0.18	0.19	0.19	0.21	0.23	0.21	0.21	0.20	0.199	0.017
	*τ*_1_	0.80	0.86	0.82	0.86	0.78	0.86	0.99	0.94	0.96	0.98	0.884	0.074
	*μ*_2_	5.42	3.96	3.94	3.69	3.63	4.02	5.47	4.20	4.22	4.08	4.264	0.617
	*τ*_2_	27.19	20.52	19.19	19.14	18.23	19.44	26.06	19.86	20.33	19.94	20.990	2.896

Interestingly, the 45° and 60° (solid blue and green lines) samples share an approximately same relaxation function, and the same for 120° and 135° (dashed blue and green lines) samples due to the supplementary relation to 45° and 60°, however, it is different for the 30° and its supplementary 150° samples. This indicates that the orientations of the two main fibres in the woven model (Fig. 5d) is between 45° and 60° (or between 120° and 135°), and the included angle *α* made by the fibres is in the range of 60°–90°, for the perfect case, *α* = 75°. Moreover, the reduced relaxation curves of the samples at the 30°, 45°, 60° orientations generally are lower than their supplementary counterparts, i.e., 150°, 135°, 120°, and this means that the samples at the distal end (30°, 45°, 60° samples) have greater relaxation effect than those at the proximal end (150°, 135°, 120° samples). This is because the proximal end is subjected to stronger force stimulation due to the heart pumping, and more elastic components of arteries are preferable distributed at the proximal end.

## Discussions

This work aims to deal with the mechanical anisotropy of arteries. The revealed microstructure of an artery clearly indicates the network of the cross-linked crimpled collagen and elastin fibres. The mechanical response of the tensile network produces the internal energy composed by the entropy and strain energy, which are contributed by straightening, re-orientation, cross-linkage, stress–strain behaviours of the biopolymers, and these contributions determine the nonlinearity and anisotropy of porcine aorta [19, 28]. The cross-linked microstructure confirms the simplified nonlinear-elastic double-layer symmetrical woven model presented by Holzapfel et al. [15], which included the angular effect of cross-linking fibres in the arterial adventitia and media. Regarding the tensile anisotropy of woven model, it was well studied in the field of the textile [29].

For the mechanical response of arteries, an intact artery behaves like smooth muscle slightly stretched about 2–20%, like elastin stretched 20–70%, and like collagen stretched over 100%, and the tension at the former two strain levels were compatible with in vivo pressures [30]. At the low strain level, the crimpled collagen and elastin are mainly stretched, and the waviness is reduced. Smooth muscle and elastin fibre are responsible for the arterial expansion, contraction, and stress distribution due to their compliance, extensibility and resilience. At the high strain level, the collagen and elastin fibre are straightened to alignment, and the ultimate stress is mainly contributed by the collagen fibre [26]. Collagen fibre serves to reinforce arteries, store energy and maintain the strength of arteries, and it is reported that the Young’s modulus and yield strength of the collagen fibril are 0.86 ± 0.45 GPa and 0.22 ± 0.14 GPa, respectively [31]. The strength of the collagen fibril is 1000 times that of the elastin fibre [32]. From Fig. 4a, we can see that the Young’s modulus calculated by the portion (<10%) of the stress–strain curve is at the low stress level, and is mainly contributed by the smooth muscle and elastin fibre, and the smooth muscle is strain rate-dependent because of its strong visco-elastic properties [33], thus, the Young’s modulus is sensitive to the loading rate. As for the ultimate stress of the circumferential sample, the failure strain is around 80%, which is consistent with the strain when collagen fibres are straightened [26]. Because the collagen fibril is stiffer and less resilient [19], and the stiffer material is insensitive to loading rate, the ultimate stress is not much loading-rate-dependent (Fig. 6b).

As for the mechanical parameters and degree of anisotropy, the circumferential samples process a better mechanical performance than the axial ones, and it can be interpreted by the stress-growth rule. This indicates that artery is stimulated by a stronger force produced by blood pressure in the circumferential direction than that in the axial, and thus the fibre mainly orientate in the circumferential direction, in this regard, the included angle *α* made by the two main fibres of the model in Fig. 5d is less than 90°. The degree of anisotropy *λ*_*θ*_ is between 0.4 and 1.0, and the range is comparable to the reported one in literature [25], which employed the ratio of Young’s modulus *E*_*θ*_/*E*_*z*_ in which the axial *z* is corresponding to the 180° samples in this paper. Due to the 2D network of the elastin and collagen fibre, their contributions to the anisotropy of arteries from both experiment and theory were investigated, but most literature experimentally documents an orthotropic anisotropic data in the circumferential and axial directions [25], and the general arterial anisotropy were only investigated by theories [20, 26]. In the sense of arterial microstructure, the anisotropy of individual component determines the arterial anisotropy at the tissue level [19], for example, the elastin was treated as an anisotropic model by the latest work [20], where the mathematical model is decomposed into two parts, one is isotropic and the other is anisotropic, i.e., *Ψ*_*elast*_ = *Ψ*_*iso*_ + *Ψ*_*ani*_. The anisotropic model characterizes more accurately the mechanical properties of the arterial wall when compared to models with a simple isotropic elastin.

Relaxation anisotropy of arteries depends on endothelial cells [34], smooth muscle, collagen and elastic fibres [24]. The role of the endothelial cells in the arterial relaxation anisotropy is mainly chemically controlled [35, 36], and isolated arteries are not so influenced with the absence of chemical agents. We here mainly discuss the influences of other components. Smooth muscle is different from the striated muscle, but they share the same sliding mechanism, namely, the muscle contraction is caused by sliding movement between the cross-bridged actin and myosin filaments connected by myosin heads, and the process is controlled by the release of Ca^2+^. Here, the histological analysis on the stained arterial sections do not show the smooth muscle, but it is easily understood that when the muscle is loaded, the sliding movement occurs, and this visco-elastic length adaptive ability enables the arteries with the greatest relaxation behaviour than the elastin and collagen [24]. Elastin and collagen fibres contribute the relaxation anisotropy at the tissue level because of their formed visco-elastic cross-linking network, and the greater cross-linking density reduces the visco-elasticity and relaxation [36] but enhances the strength and toughness [37]. Besides, in the deformation process of the arteries, the straightening of the crimpled collagen and elastin fibre or the unfolding of protein domains also contributes to the relaxation. It is worth mentioning that contrary to common sense, the collagen or elastin fibre reorientation seems not a primary source of their visco-elastic properties [38], instead, the relaxation process within the collagen fibres or at the fibre-matrix interface is speculated to be responsible for the arterial relaxation.

In summary, we have made a comparatively comprehensive examination of arterial anisotropy compared to the conventional data in the circumferential and axial directions, in particular for the relaxation anisotropy. However, the limitation is that the isolated artery is cut into open and the residual stress is released.

## Conclusions

In this paper, we have made two sets of uniaxially tensile experiments on the anisotropy of arteries by tensioning multi-directional stripped aortic samples. The Young’s modulus, ultimate stress, the degree of anisotropy, and relaxation anisotropy of the stripped samples were obtained. The Young’s modulus is more sensitive to the loading rate than the ultimate stress, but the degree of anisotropy is not loading-rate dependent. Due to the preferable fibre distribution in the circumferential (90°) sample, it has the greatest mechanical properties and the smallest stress relaxation, whereas the axial (180°) sample has the smallest mechanical properties and the greatest relaxation, and the mechanical behaviours of the other samples are in-between; moreover, the mechanical behaviours monotonously increase as the samples’ orientations change from the axial to the circumferential directions. Compared to the previous work, the present study examines the arterial anisotropy in multi-directions, and is very helpful to understand the mechanical anisotropy of arteries.
